# Additive Manufacturing for Repair: Continual Construction Through Bio-Based Materials

**DOI:** 10.1089/3dp.2023.0344

**Published:** 2025-04-14

**Authors:** Mette Ramsgaard Thomsen, Paul Nicholas, Ruxandra-Stefania Chiujdea, Stine Dalager Nielsen, Konrad Sonne, Carl Eppinger

**Affiliations:** Department of Architecture and Technology, Centre for Information Technology and Architecture, Royal Danish Academy—Architecture, Design, Conservation, Copenhagen, Denmark.

**Keywords:** additive manufacturing, circular design, repair, maintenance, 3D printing, biopolymer composites, digital design methods

## Abstract

The article asks how additive manufacturing for the circular bioeconomy can create the foundation for rethinking the architectural axioms of permanence and durability, instead moving us toward a new ideal of renewability and repair. It presents a case study into additive manufacturing for repair through the 3D printing of biopolymer composites. This case study connects machine vision-based surveying of damaged panels with repair through conformal 3D printing. This deployment of bio-based materials aims to enable additive manufacturing as a method for disrupting the sharp delineation between fabrication and repair leading to new practices of continual construction. With point of departure in our bespoke systems for 3D printing and unique biopolymer composites, we examine how their particular material characteristics allow for material adhesion and buildup and how novel methods for iterative 3D printing can support design integrated strategies of repair. As part of this process, we include the sociotechnological dimension, as human-in-the-loop decision-making becomes part of the material surveying regimes necessary for damage detection. The article demonstrates processes of repair through three repair actions that address different kinds of damage.

## Introduction

The push to develop more sustainable building practices is challenging architecture, engineering, and construction to develop new design methods that combine circular and bio-based practices. Large-scale efforts such as the European Green Deal^1^ challenge existing linear “take-make-dispose”-based models of resource deployment by ideating methods to keep products and materials at their highest value across multiple life spans. A fundamental part of this effort is to devise methods of innovating the use of waste-sourced, bio-based material derived from agri- and silvicultural production streams. By being inherently renewable and acting as a carbon store, these material classes upcycle underutilized waste and residues otherwise composted or burnt for energy.^2,3^ Biopolymer composites are a particular instance of these materials and are receiving much attention for their versatility and ability to be tuned to particular performances. While this material class is new, biopolymers, bio-composites, and bio-based polymers have a deep history preceding fossil fuel polymers, their biocompatibility and the ability to design their specific lifespans through planned decay are finding new applications in biomedicine and the food industry.^4^ In architecture, the same properties are understood as important avenues for creating novel material systems that are compatible with the principles of the circular bioeconomy. The ability to design systems that upcycle waste-sourced, bio-based materials into building components allows us to imagine the built environment as participating in the larger ecological cycles of growing and harvesting materials. The wider societal ambition for these cycles is to both *close* and *slow* the resource loops for them to become effective carbon stores. However, the lesser strength ratios and inherent biodegradability of biopolymer composites challenge this goal of durability in building practice. Therefore, there is a need for novel design systems that slow down resource loops by retaining bio-based components using methods of continued repair and maintenance.

This article presents a novel fabrication paradigm of continual construction deploying biopolymer composites. It hypothesizes that repair methods for biopolymer composites can be performed through responsive aperiodic acts of additive manufacture that takes both the inherent behaviors of the material system as well as the fabrication restraints into account. As a novel extension of the digital chain, we examine how additive manufacturing for repair can generate new continua between fabrication and repair. The hypothesis is tested through the experimental case study of a set of 3D printed bio-composite panels that aggregate continuous materialities as they are repaired with varied versions of waste sourced biopolymer composites. The article outlines the workflow of these novel methods by detailing the iterative processes of material surveying, repair strategy identification, reprint path adaptation, and conformal printing.

## State of the Art

Additive manufacturing is an emerging fabrication method for biopolymer composite materials in architectural research. Biopolymer composites are designed materials that compose multiple material streams. They combine a binder—the biopolymer—with a solvent, as well as fibers and fillers to create a composite material.^5^ Examples focus on the extrusion of slurry-based materials for the fabrication of architectural scale elements. Efforts have been placed in understanding the interdependent relationship between 3D printing parameters and material properties, or to control fabrication and geometrical parameters^6^ and to create porous spatial lattices.^7^ Further research investigates material behavior following the printing process, specifically the material’s shape response to the dehumidification that takes place during drying processes.^8^ In Rossi et al.,^9^ this behavior is tracked and modeled to predict shape response in advance so that it can be incorporated into a design modeling workflow.

The shape response is driven partly by the material’s hygroscopic properties, as both the polymer and fillers are able to absorb and release moisture, but it is also dependent on the 3D print toolpath design. Materially sparse printing strategies ensure a high surface-to-volume ratio and porosity at the element scale that facilitates airflow and therefore consistent drying behavior. These fabrication-led design drivers are important in the scaling up of the components as they interface with other design factors including structural integrity, assembly logics, and force flows through assemblies. Research has been targeted toward architectural elements such as columns,^10,11^ grid shells,^12^ interior freestanding wall,^13^ interior wall claddings,^3^ and responsive screens.^14^

While this research examines biopolymer composite behavior within the interior, our research goes beyond state of the art by investigating biopolymer composite components within external conditions. We have previously reported the mechanical characterization of varying compositions of our material recipe^15^ as well as the use of machine vision-based monitoring to track the ongoing behavior of externally exposed panels and automated recognition of shape change. Furthermore, in 2023^16^ we have reported the use of Machine Learning (ML) to predict parallel biopolymer composite recipes and the impact of post print drying on geometric tolerance.^17^

In this article, our focus is on how additive manufacture through 3D printing supports methods of continual construction in which processes of fabrication are extended through reparatory action. We work with panel elements that have been exposed to higher levels of humidity and direct weathering through solar exposure, precipitation and wind. In these conditions the inherent shape-response behavior is greatly amplified while weathering accelerates degradation and damage. These responses expose the greater material fragility and catalyze damages, initiating the need for repair.

Repair and maintenance are mundane aspects of building life and are conventionally separated from the processes of designing and building architectural structures. Repair actions can take the form of common processes of renovation or as specialized processes of preservation. Both seek to restore performance and conserve the integrity of the building mass. Repair actions belong to an established catalog of generic strategies that are adapted to the specific given damages. This is done through careful selection of suitable materials that can bond with the damaged building elements and are aligned with local material behavior.

Central to repair are the processes of surveying. These connect inspection of the material condition to the diagnosis of degradation and damages and support decision-making for the modes of intervention needed and their urgency. Practices of surveying can be human-driven and local automated and remote. It is common to use handcraft tools such as crack gauges to measure cracks, sound hammers to check adhesion loss of tiles, or resistance meters to measure the moisture content in wood.^18^ When more data are required, advanced tools such as laser scanning and thermal imaging are employed.^19^ For inspection of structures harder to access by humans, automated surveying methods are used such as robotic systems equipped with computer vision to detect cracks on bridges, dams, or silos.^20^

**FIG. 1.**
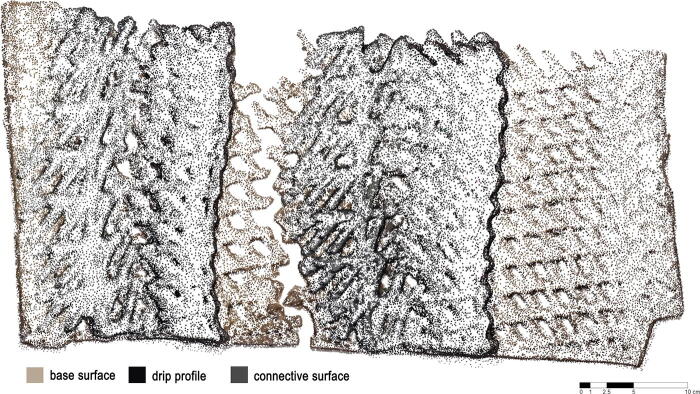
Point-cloud of weathered state of 3d printed biopolymer composite panel for architectural weather screening. The registration of material colour differentiation aids the automatic identification of panel features.

In our research, we connect these surveying practices directly to Additive Manufacture (AM)-based repair-driven fabrication processes (Fig. 1). AM is already utilized as a repair technique in a broad range of applications. It is applied at object scale, for instance, for fixing household objects^21,22^ or conserving building ornaments and statues.^23,24^ In this case, 3D printing is used as a strategy to replace missing fragments by using an alternate 3D printable material such as Poly Latic Acid (PLA) or gypsum. At a larger scale, 3D printing is used to restore building components^25,26^ or mend damaged road infrastructure.^27^ In both these instances, registration methods such as photogrammetry are employed to develop 3D-models for identifying areas in need of repair.

In parallel to these technology-driven explorations, researchers have deepened conceptual understandings around repair. Steven Jackson’s concept, *Broken World* Thinking,^28^ is becoming a key framework for describing the fragile disposition and resulting intensified relation between materials, technologies, humans, and environments. According to Jackson, who builds on earlier studies by Leigh Star and Bowker,^29^ *Broken World Thinking* regards “an appreciation of the real limits and fragility of the worlds we inhabit-natural, social, and technological.”^28^ Here, fragility—and the resulting need for maintenance and repair—is not the exception but the norm.

## Methods

**FIG. 2.**
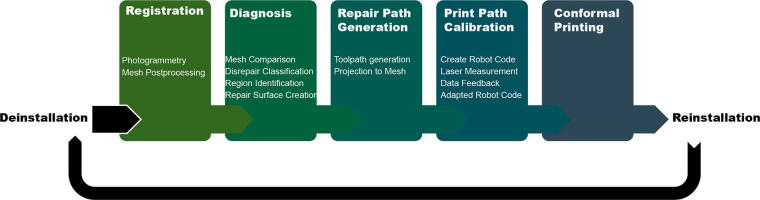
Diagram of overall repair workflow.

In our experiment, we design and repair a series of architectural weather screen panels. The repair method creates a novel workflow that connects machine vision-based surveying to diagnosis and repair strategy identification, repair path design, and adaption and finally the conformal printing of repairs (Fig. 2). The repairs are made using the same biopolymer composites as the original panels.

### Recipe variation and heated 3D printing of biopolymer composites

Our biopolymer recipe combines a protein-based biopolymer (a collagen glue), with cellulose fillers derived from various waste stream drawn from agricultural and anthropogenic production.^30^ The collagen glue binder is mixed with cellulosic fibers that act as reinforcements and fillers. The fibers are the residues of local agricultural production. They include bark and wood flour originating from the timber industry and cotton sourced from end-of-life linens and clothing. In previous work, we have reported the recipe and evaluated the performance of recipe variations. Here, the rheology and tensile properties of the recipe variations using distinct fibers and varied quantities has been assessed.^15^

**FIG. 3.**
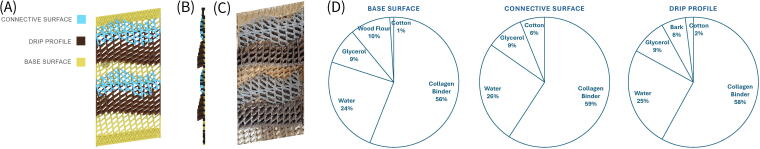
**(A)** Plan, **(B)** principal section, **(C)** printed panel, and **(D)** the three recipe variations deployed in the panels.

The weather panels combine three such variations of the base collagen recipe each mapped to distinct features: the base surface, the drip profile, and the connective surface (Fig. 3A, B). The base surface (first two layers) includes 10% wood flour and 1% cotton. Here, the addition of a small amount of cotton improves the rheology of the material allowing a consistent material flow. The drip profile is printed with 6% bark and 2% cotton. The high bark content changes the color of the material aiding the automatic identification of this feature in the photogrammetry data. The higher cotton content makes it possible to print with a 30**°** cantilever. The connective surface is composed with 6% cotton. This gives the material a strong tensile performance deployed to connect the drip profile back to the base surface while at the same time creating a distinct blue color that again is easy to identify in the photogrammetry registration.

**FIG. 4.**
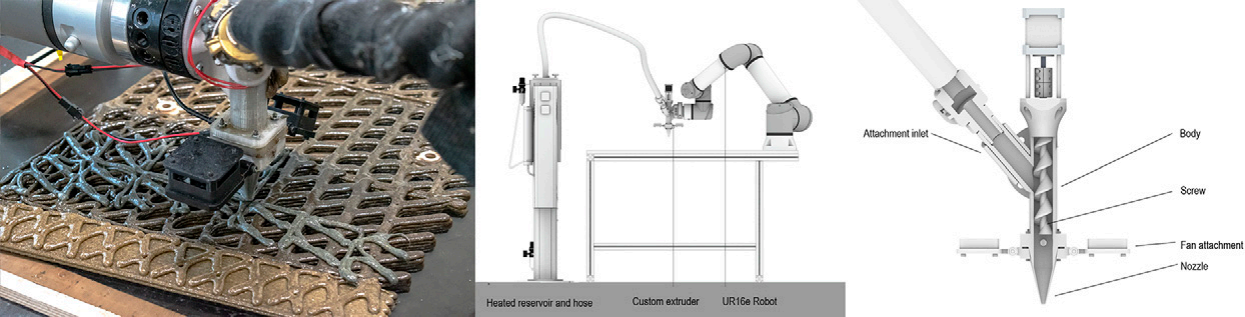
Photograph, close-up detail of extruder head, and diagram of the 3D printing system. The heated material is pneumatically fed to a custom-designed extruder via a heated reservoir and hose.

This material’s 3D printing necessitates a heated system. Heat, in combination with water and polyols, gives the biopolymer composite thermoplastic-like properties that allow it to flow (Nicholas et al. 2023a), The design of the printing system (Fig. 4)^31^ incorporates key considerations regarding material, viscosity, and temperature control. The custom-built extruder is designed for robustness against abrasions from the fillers, to reduce the possibility of the collagen bonding with the extruder, and for easy cleanability. The extruder incorporates a Nema 17 stepper motor, DS18B20 temperature sensor, and 2 × 14 v 40 mm DC fans, with the extruder body, screw, nozzle, and attachment points custom-made. The biopolymer composite is pneumatically pumped to the extruder from a separate reservoir. The reservoir and the hose are both heated to 67° C, optimizing material flow within the system. The extruder is unheated, and fans mounted to the nozzle immediately cool the material post extrusion.

### Machine vision-based surveying: Registration through photogrammetry

Our method integrates a photogrammetry-based approach for material surveying. The porous geometry is highly self-obscuring and ill-suited to fixed position lidar scanning. Instead, the versatility and scale appropriateness of the handheld Scaniverse tool^32^ has shown to give best results. The photogrammetric registration of the single panel obtains a mesh model that includes surface geometry and color data. The photogrammetry is performed in a controlled indoor environment with stable lighting conditions. To scale the mesh, we include four registration spheres at known positions within the photogrammetric registration. The automated scale correction identifies the center of these spheres and scales the mesh accordingly. An initial registration is made directly after printing and subsequent registrations are made when repair is required.

### Diagnosis and repair strategy identification

**FIG. 5.**
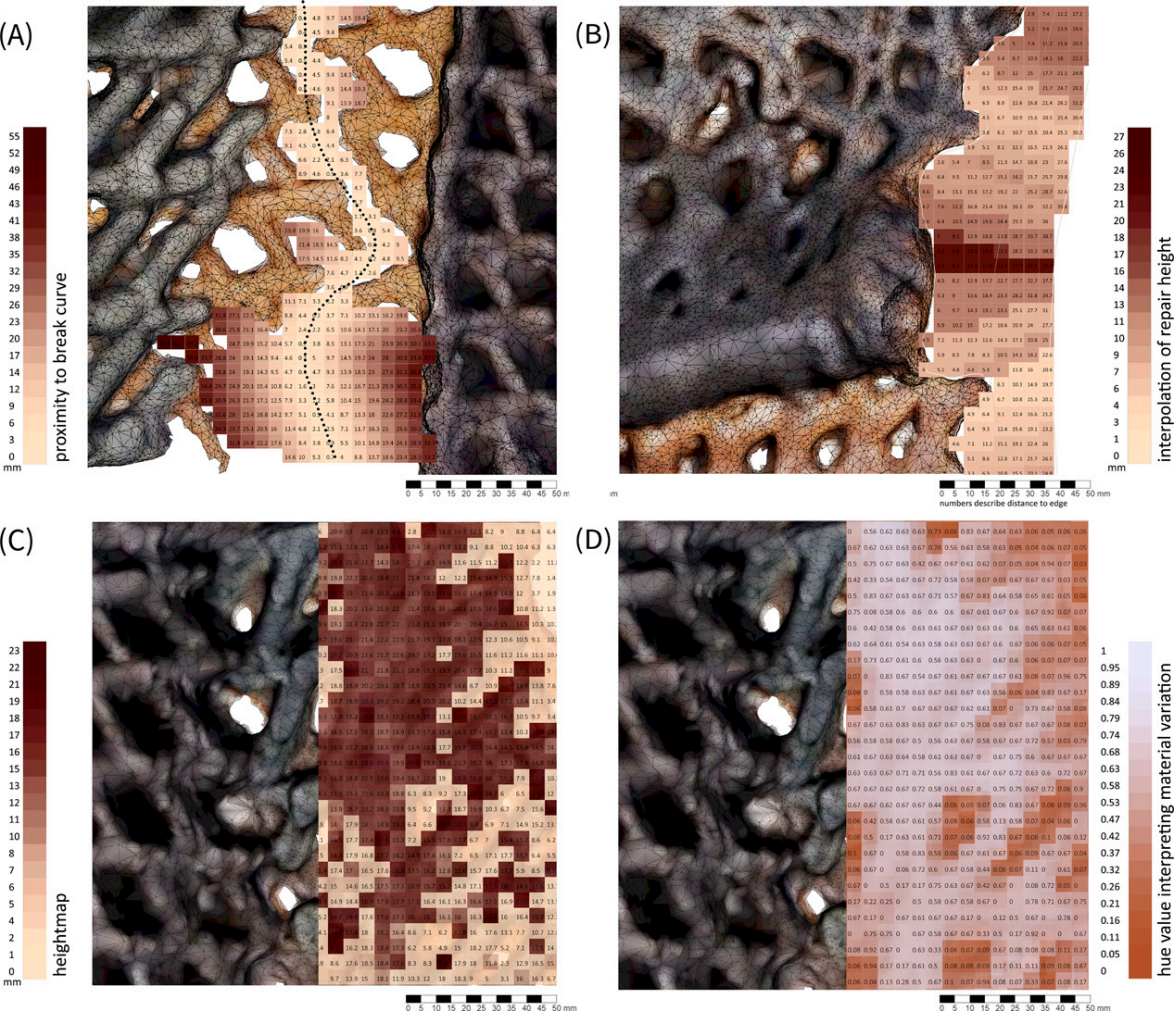
Data-driven modes of material survey are used to establish **(A)** the relative planimetric boundary difference (regions of shrinkage), **(B)** curve proximity (cracking), **(C)** z-height (to diagnose loss of definition), and **(D)** hue value (for identifying materially differentiated print regions).

The diagnosis of disrepair is based on a comparative method. The two mesh models are compared to determine regions that require repair as well as the types of disrepair. Comparative modes include processes for establishing the relative planimetric boundary difference, curve proximity, z-height difference, and hue value (Fig. 5). These data are used to diagnose three types of disrepair that occur as a result changes in humidity and solar radiation: (1) shrinkage, which takes place through the contracting and warping of the material, (2) cracking, where panels separate into multiple pieces, and (3) loss of definition, where previously sharply defined geometries soften as result of swelling.

*Regions of shrinkage* are identified by computing the relative boundary difference as a surface an defining the region requiring infill. The process is applied individually across the four edges. *The crack boundary* is identified by defining a central crack-line and identifying mesh vertices that lie on the boundary of the crack based on proximity to the crack-line. The boundary is computed as an infill surface. *Loss of definition* is identified through a filtering of the mesh node z-height and color hue. Regions requiring redefinition are limited to specific features that occur at the top of the panel and are printed using a color differentiated recipe. These regions are therefore firstly identified on the basis of their hue, and then evaluated on the difference between the z-height of the two mesh models.

### Repair path design

These diagnostic methods are linked to case-specific repair strategies, as detailed below. In each case, the regions are infilled with 3D-printed geometries that reestablish the integrity and performance of the panel. The zone of repair extends beyond the damage to ensure bonding between repair and panel. The reparatory print paths populate the calculated surfaces. Initially designed as if on a flat print bed, they are then projected onto the photogrammetric mesh surface to interface with the existing print.

### Conformal printing and print path adaption

**FIG. 6.**
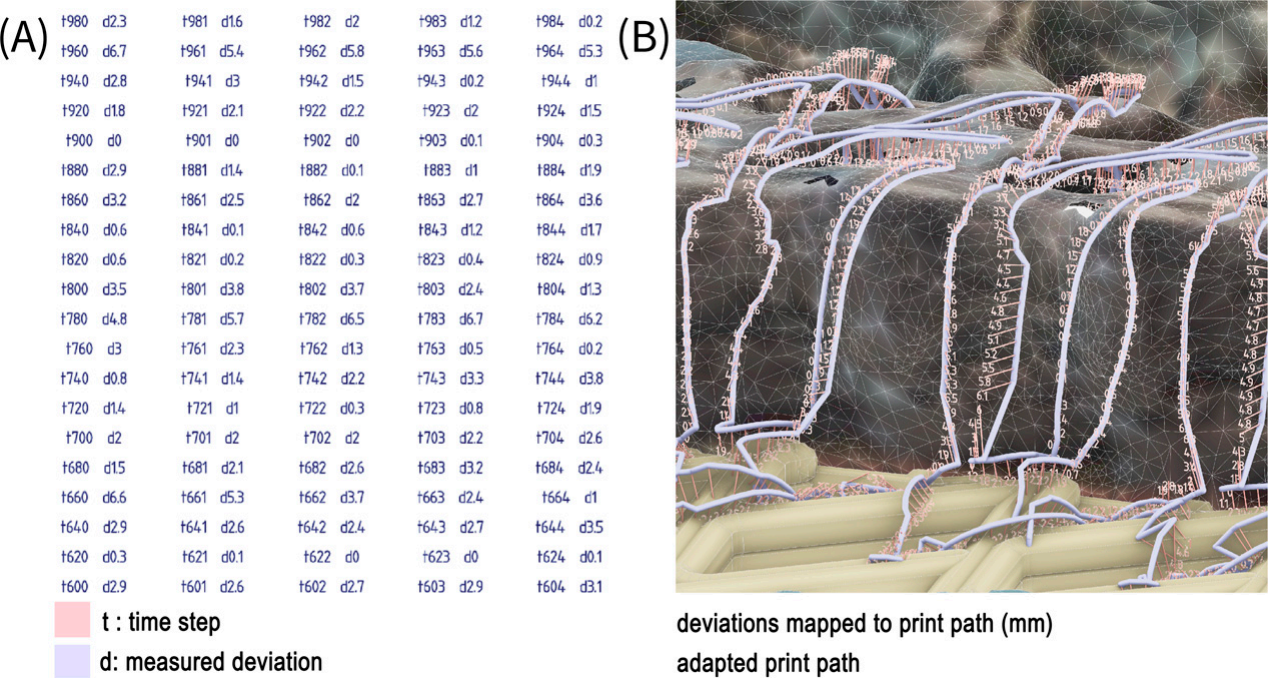
**(A)** Adaptation of conformal print path is undertaken by attaching a laser single point laser measure to the robot and running the projected toolpath with corrective measures taken at evenly time-stepped intervals corresponding to ∼2 mm. **(B)** Corrective measures are stored in a database.

The projected print paths are used for conformal printing of the repairs onto the panels. The thermoplastic-like behavior of the biopolymer allows us to reprint new material layers on top of existing prints with a very high level of interlayer bonding, creating a materially consistent repair. To achieve sufficient bonding, a constant distance between the nozzle and the underlying print surface needs to be maintained. During preliminary experiments testing the accuracy of the process, we found that photogrammetry mesh did not provide sufficient accuracy. To correct the toolpath, we used a single point laser measure. The laser measure is attached to the robot and the projected toolpath is run with measures taken at evenly time stepped intervals corresponding to ∼2 mm. Feedback from this time-stamped dataset is then re-correlated to the robotic toolpath and used to locally adapt the z-heights of robot target points (Fig. 6).

### Case study design

The project examines repair through the making of an external weather screen, which represents a use case for 3D printed bio-based materials. Weather screens are minimal facades that screen the building from wind and rain while remaining lightweight, open, and materially efficient. The panels are designed with specific material and geometric features that enable them to achieve different performance and fabrication-driven criteria. First, the panels are printed with high level of porosity to minimize material use and modulate weather while allowing airflow and visibility to the outside. Second, the panels are geometrically and materially differentiated. Each panel is designed as a multi-material composite strategically varying the recipe to achieve graded performances. The panels are made of three layers: a base layer, printed with wood flour, cotton fillers, and fibers; a connective layer, printed with bark and cotton; and a sacrificial overhang layer, printed with bark, which acts as a drip profile forcing water off and away from the surface. The three layers are printed to create one consolidated composition.

**FIG. 7.**
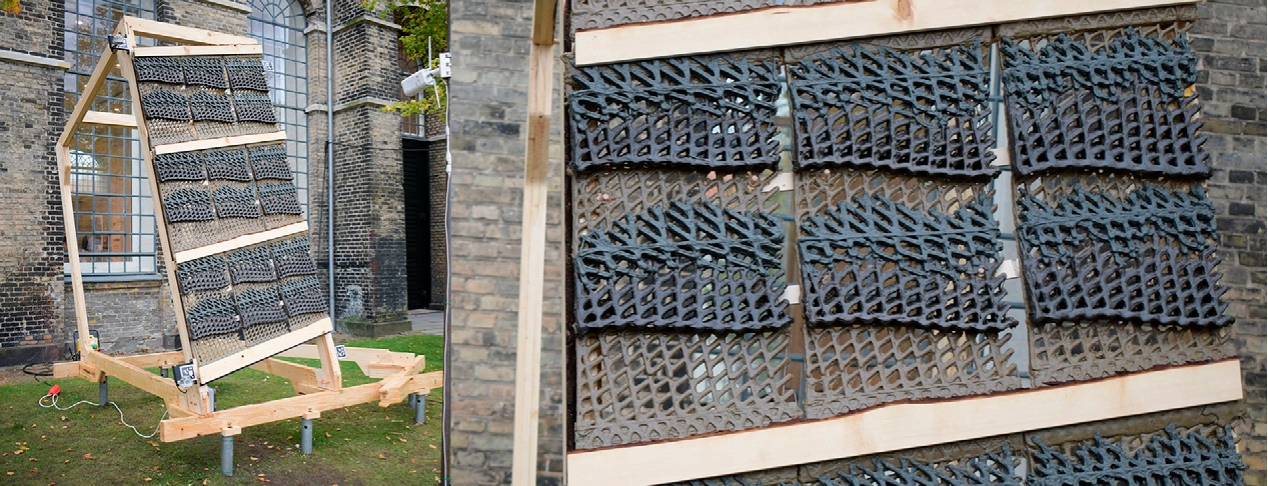
Biopolymer panels tested as an external weather screen installed on the observatory rig. The individual panels measure 700 × 350 mm.

The panels were mounted on an observatory rig for 4 months (Fig. 7). Prior to being mounted, each panel is 3D scanned to capture its initial “as printed” state. Information from this scan becomes a key reference for subsequent diagnostic and refabrication processes. The observatory rig is exposed to typical wind and environmental loads in Copenhagen from July to October. This period included strong gusting winds of up to 36 m/s and highly differentiated humidity levels ranging from 33% to 95%.

**FIG. 8.**
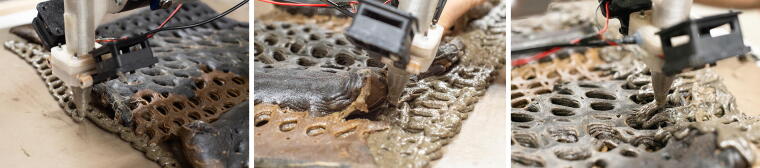
Repairing the damaged weather screen panel through a combination of flat-bed and conformal 3D printing.

This regime sets up multiple modes to track material change and register damage. As damages are registered, the panel is removed from the rig and taken to the refabrication lab. Here, the panel is manually cleaned and dried to remove excess moisture in the material. The panel geometry is then registered at high resolution using photogrammetry. The resulting 3D mesh is compared with the as-printed state scan to drive diagnosis and select repair actions. The definition of reparatory tool paths is designed to match the geometric language of initial panel design and adapted by the designer to the specific site of damage (Fig. 8). Based on our experiments and the behavior of our material, we have identified three central repair actions which are described in the following section.

## Results: AM for Repair in the Case Study of a Biopolymer Composite Weather Screen

### Repair action 1: Over-weaving

As the panels are placed outside, they are exposed to fluctuations in humidity. The material system reacts to these changes by warping, which in turn creates a reduction of the overall plane surface area. This reduction is seen as a shrinkage of the panel edge, opening gaps between the panels that allow unmodulated weather (rain and wind) to enter through the weather screen. The warping behavior is affected by the varying porosity in the panel as well as its layering. In materially dense areas, where porosity is low and material build up is high, warpage is more accentuated.

**FIG. 9.**
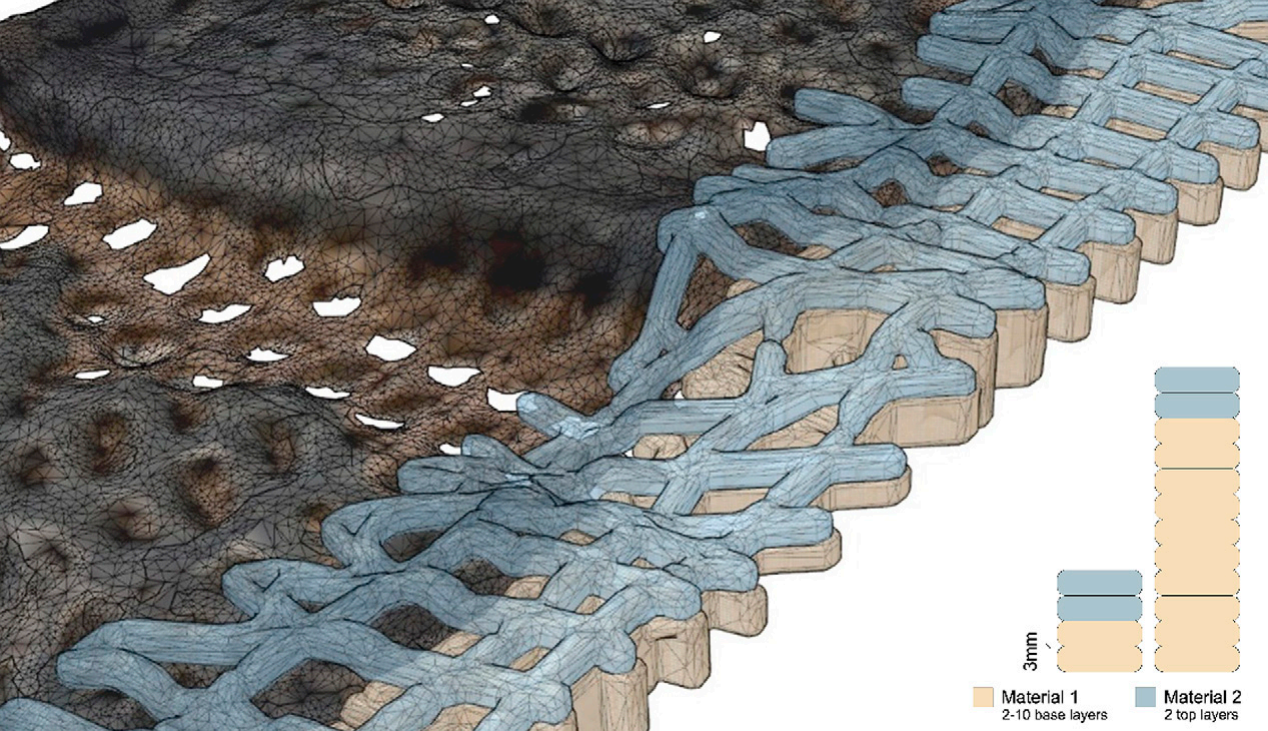
Diagram of over-weaving repair action. The reparatory print path restores the region requiring infill: The base layer infills the relative boundary difference while the second over-weave layer creates a connective lattice. The process is applied individually across the four edges.

To reestablish the edge, the over-weaving repair action includes both flatbed printing and conformal printing (Fig. 9). The high-resolution photogrammetry registration is analyzed to establish the transition between flatbed and conformal printing as well as to precisely calibrate the toolpaths to the panel surfaces. The repair print is structured across two layers. The first base layer builds up the missing base surface while the second over-weave layer creates a connective lattice. The base layer varies in height to fit with actual geometries of the weather panel. The over-weave layer connects the base to the weathered panel by spanning across the two. This connection is further consolidated by identifying perforations in the initial print and filling them to create solid cohesion. Furthermore, at a material level, the heat and the moisture of the freshly printed material activates the thermoplastic behavior of the weathered panel enabling self-adhesion. Finally, the layers are printed using material compositions that match their distribution in the initial print to achieve continuity in material performance.

### Repair action 2: Patching

The second repair action addresses breakages within the panels. The combination of humidity change and wind can cause the panels to break. Breakages can be complete, where panels break across the entire surface, or partial, where parts of panels break off. As with shrinkage, the impact of breakage is to open large gaps that allow unmodulated weather to enter. The varying material density of the panel affects the breakage with areas with higher porosity and less layer depth create moments of structural weakness. During the 4-month exposure period, we detected breakage through daily human inspection. Following breakage, panels are dismounted and broken off parts are collected. They are manually cleaned, dried, and registered using high resolution photogrammetry. The resulting point clouds are used as the basis for a comparative analysis by which to determine break lines and missing areas.

**FIG. 10.**
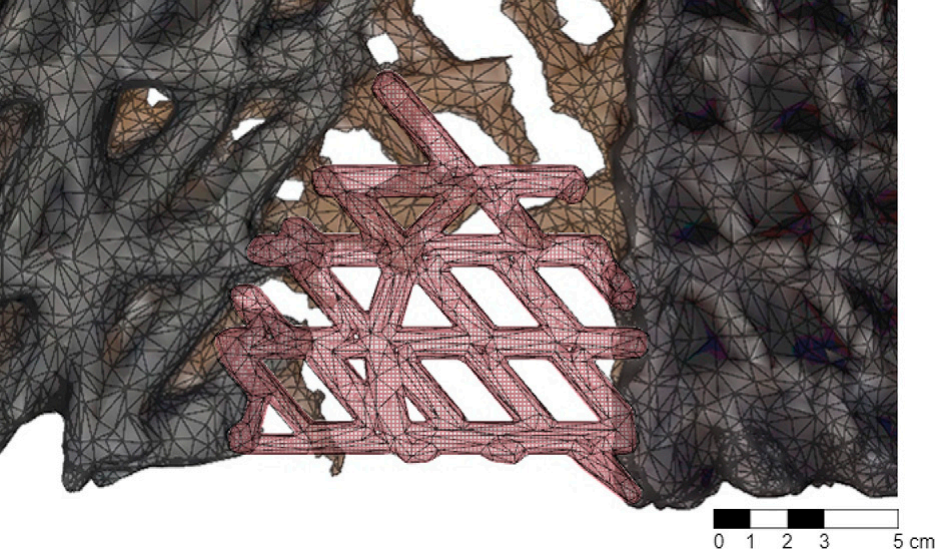
Diagram of patching repair action. The reparatory print path restores the panel: missing regions are infilled through flatbed printing of a patch and a connective lattice is conformally printed to stretch across the entire patch or, in wider areas, connect either edge of the patch.

The objective of the patching repair action is to define the geometry of the missing areas and reconnect panels across crack lines (Fig. 10). As with the over-weaving repair action, the patch action combines both flatbed printing and conformal printing. The printing is structured across two layers. A first base layer builds up the surface in missing areas to while a second connective lattice binds the patch to the weathered print printing into panel perforations to create solid cohesion. Depending on the size of the breakage, the connective lattice can either stretch across the entire patch or, in wider areas, connect either edge of the patch to the weather panel.

### Repair action 3: Re-detailing

The third repair action addresses loss of detail within the panels. As panels are exposed to external conditions, the acute increase in humidity causes a one-way shape change in the hygroscopic materials in the form of a swelling and loss of material resolution. The swelling behavior is particularly seen in the sacrificial overhang that act as drip profiles and therefore directly interface with precipitation while areas that are shielded by the overhang demonstrate lesser swelling. The swelling changes the shape of the drip profile and makes it harder to lead water away from the panel surface.

**FIG. 11.**
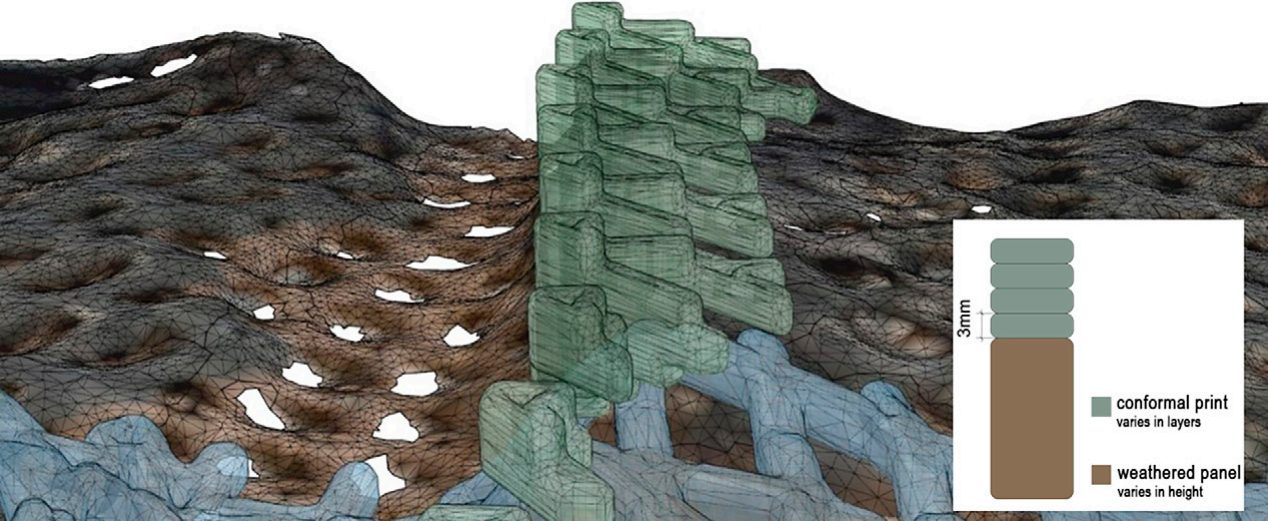
Diagram of re-detailing action deploying conformal printing to reestablish the drip profiles onto the panels.

The swelling behavior is recorded through human inspection when a panel is taken down. High-resolution photogrammetry registration is used to analyze the extent of the swelling behaviors and geometric deformation. The reprinting process uses design information from the initial panel design to reprint the drip profile onto the weathered print (Fig. 11). The re-detailing print action is achieved using a multilayer conformal printing strategy. By matching the geometric and material composition of the weathered print, the functionality of the drip profile is reestablished.

## Conclusions

The article showcases results that advance the field beyond the state of the art by creating a foundation for a new understanding of architectural materiality as essentially malleable affected by time, use, and environmental impact drawing in processes of continual care and repair. The three repair actions demonstrate the use of additive manufacturing for the repair of bio-based building components. The parity between the processes of fabrication, the identical material compositions, and the ability to instrumentalize the thermoplastic-like behaviors of our biopolymer composites means that we can ideate new methods of continual construction for bio-based materials. These methods rupture the otherwise clear delineation between fabrication and repair, suggesting novel ways by which to engage true cascading in the circular bio-economy. While these methods can scale, they are developed here as prototypical methodologies that suggest novel ways of extending the life spans circular bio-based materials. While the research reported in this article remains prototypical and remains framed by the lab environment, the larger vision guiding their developments is aimed toward on-site fabrication processes that locate and situate the reparatory actions. Here, buildings clad with assemblies of panels are monitored and damages to individual panels identified. These single panels are then temporarily removed and taken to an on-site fabrication facility where repairs would take place.

The evolving conceptualization of these AM for repair processes therefore includes practices of temporary replacement and resurrection of panels. A central contribution in this novel methodology is the feedback loops between the processes of surveying, diagnosis, and refabrication. Where repair practices otherwise understand these as separate and disconnected acts, our method develops ways of integrating these into a comprehensive AM-based repair method. Our approach demonstrates a broadening of material surveying and repair processes to consider the high malleability and resulting responsiveness to changing environmental conditions of our material system. In doing so, it connects human, technological, environmental, and material agencies and explicitly places the human in the loop within this circular design approach.

Our concern for repair foregrounds the fragility of a biogenic architecture. Here, the different strength ratios and durability of biopolymer composites are seen not as weaknesses but as formative constellations. In this case study, we see these properties as opportunities to recast AM as an iterative process of reprinting. As such, we return to Jackson’s *Broken World Thinking* casting repair not only as preservative but also as fundamentally transformative positioning fabrication systems within an extended understanding of endurance, circularity, and lifespan, all three new key terms within a post-Anthropocene architecture.

Further research will ideate how the methods of repair can be reiterated and what role data can play within these cycles of repair. The AM-based method presented here is inherently iterative. Repair actions will be continually called and the layers of over-weaving, patching, and re-detailing will superimpose. Our future research aims to understand the performative and architectural consequences of these repairs and behaviors as they build up over time.
